# Role of traditional knowledge in the COVID‐19 battle

**DOI:** 10.1111/jwip.12220

**Published:** 2022-03-06

**Authors:** Kunle Ola

**Affiliations:** ^1^ Thomas More Laws School (Brisbane) Australian Catholic University Banyo Queensland Australia

**Keywords:** COVID‐19, intellectual property, traditional knowledge, traditional medicine

## Abstract

This article addresses the role of traditional medicine in the corona virus (COVID‐19) battle. It situates traditional medicine within the traditional knowledge and Intellectual Property discussion. The paper discusses the increased gravitation towards using traditional medicine but also identifies the existence of scepticism towards its use. Indigenous communities make up a large portion of the traditional medicine community and are therefore called upon to play their role in the fight against COVID‐19. The battle is real, the coronavirus ground is an intersecting one with multiple, plausible solutions. Traditional medicine has a role in this battle and this article advocates for collaboration. It is a clarion call for all stakeholders to join hands to fight the virus. It advocates inclusion rather than exclusion.

## 1 INTRODUCTION: THE WAR AGAINST COVID‐19

Scientists are working around the clock, waging war against COVID‐19 to defeat the virus and address the devastating effect of the pandemic.^1^ Waging war is hard because of the impact it has on everyone.^2^ The impact is felt by everyone including from the Generals planning the war strategies, to the fighters on the field, to the families left at home, panic‐stricken and apprehensive as to whether their loved ones would come back home in one piece; to the entire nation that often bears the social, economic and political impacts of war, such as recession, physical destruction, diseases and years of unrest. The COVID‐19 war is no exception with national and international governments faced with planning COVID‐19 strategies. While scientists continue to work on developing vaccines, healthcare workers are on the frontline treating thousands of sick people, and they are concerned that they may contract the virus and spread it to their loved ones.^3^ The economies of the world are plummeting. Nations that had achieved decades of economic stability have been hit hard by this pandemic. After almost three decades, Australia known as ‘the lucky country’ has been pushed into economic recession by the pandemic.^4^ The New York Times noted that ‘The battle against COVID‐19 has ended an impressive growth streak, but other challenges will make it hard for “The Lucky Country” to match its past success’.^5^ The IMF described the impact of the pandemic as the worst since the Great Depression of the 1930s and that the pandemic has plunged the world into a ‘crisis like no other’.^6^ Social life is dying due to COVID‐19 restrictions and all of these are clearly impacting nations, families, communities and individuals.^7^

### 1.1 WHO declares a pandemic

The coronavirus outbreak was declared a Public Health Emergency of International Concern (PHEIC) on 30 January 2020 by the World Health Organisation. On 11 February 2020 the disease was named COVID‐19.^8^ By 11 March 2020 it had gone around most parts of the world and was declared a global pandemic due to the alarming levels of spread and the severity of the disease.^9^ As of June 2020, the top 10 countries affected with the virus were the United States, Brazil, Russia, India, UK, Spain, Peru, Chile, Italy and Iran.^10^ In the United States, New York City was worst hit and the demand for ventilators spiked.^11^ In a desperate effort to find a cure, unconventional strategies had to be deployed. General motors and Ford known for making automobiles began making ventilators.^12^ In a similar desperate measure, the United Kingdom through its campaign for Rapidly Manufactured Ventilators System (RMVS) offered to indemnify ventilator manufacturers against possible Intellectual Property (IP) claims.^13^ Information began spreading that Hydroxychloroquine could cure COVID‐19 and this led to a rush for the drug.^14^ President Trump called the drug, the ‘game‐changer’.^15^ Desperate for the drug, he threatened retaliation against India if they refused to ship the drugs.^16^ Prime Minister Modi lifted the ban and the drugs were shipped.^17^

At the onset of the outbreak, some people made light of the spread of the virus until WHO declared it a pandemic.^18^ The hospitals became full and the morgues could not take in more dead bodies. Fear creeped in, panic buying filled the air and then the restrictions set in causing an unprecedented global lock‐down.^19^ Many people began resorting to herbal remedies, natural roots and foods from plants such as garlic, ginger, turmeric, onion, cucumber, broccoli and other naturals.^20^ The idea was that these natural products are good at boosting the immune system and would enable the body fight off the virus.^21^

Medical science has enabled improvements to the general healthcare and wellbeing of the average person.^22^ This is evident from the invention of pharmaceutical products like penicillin, polio vaccination and malaria drugs which have prevented millions of deaths.^23^ The world is indebted to medical science for the strides that have been taken and the mammoth achievements in the health care sector.^24^ This pandemic calls to the fore the importance of medical science. It has also drawn the world's attention to an increased gravitation by many to herbal remedies and organic/natural products.^25^ Herbal remedies have become widely accepted and are also referred to as traditional medicine.^26^ Kamboj opines that

Herbal medicine is still the mainstay of about 75–80% of the world population, mainly in the developing countries, for primary health care because of better cultural acceptability, better compatibility with the human body and lesser side effects. However, the last few years have seen a major increase in their use in the developed world. In Germany and France, many herbs and herbal extracts are used as prescription drugs and their sales in the countries of European Union were around $ 6 billion in 1991 and may be over $ 20 billion now. In USA, herbal drugs are currently sold in health food stores with a turnover of about $ 4 billion in 1996 which is anticipated to double by the turn of the century.^27^

Herbal products are natural and are made up of herbs, roots, leaves, berries and flowers which contain active ingredients that are helpful to the body. The use of traditional medicine is still contentious and viewed with scepticism in some quarters, but its use has become widely accepted.^28^ This article is a clarion call to the world to explore and optimise all available opportunities to combat this pandemic.

As the world gradually attacks the deadly virus, the coronavirus statistics of new cases and recorded deaths is alarming.^29^ The social, economic and health havoc it has caused will take years to fix and the earlier this virus is brought under control, the better for the world.^30^ No doubt, the virus will be subdued because humans are wired to subdue their environment.^31^ Inventions like the printing press, light bulb, airplanes, personal computers, automobile, clock, telephone, refrigeration, camera and vaccines speak to the ingenuity and tenacity of human beings to confront challenges and find solutions to current and future problems. These innovations have no doubt increased our quality of life and made possible what was hitherto impossible. Humans are once again at the cusp of another problem and this time, it is the coronavirus. Early in 2020 the thoughts would have been who will develop the vaccine? Companies like Pfizer, BioNTech, Moderna, Novavax, AstraZeneca, Sionpharm and Gamaleya have developed vaccines.^32^ One thing is sure in the battle against COVID‐19, everyone is a potential victim and therefore everyone's happiness counts. To win this war, collaboration is key, and it triumphs over isolation, just as the collective interest should take priority over individual interests.^33^

### 1.2 Collaboration is crucial in this battle

In the Art of War, the author lays out war strategies and amongst the strategies, he discusses the nine types of grounds including the ground of intersecting highway.^34^ Sun Tzu commenting on this ground noted that ‘he who occupies it first has most of the Empire at his command’ and he advises that when on such a ground the best strategy is to ‘join hands with your allies.’^35^ To combat this virus, NIH has noted that ‘By bringing together experts with complementary skills, knowledge and experience, NCATS helps projects cut through operational roadblocks to contribute to new knowledge about COVID‐19 and the virus that causes it.’^36^ Through the Open COVID Pledge, organisations around the world, International group of researchers, scientists, academics and lawyers are working to ‘accelerate the rapid development and deployment of diagnostics, vaccines, therapeutics, medical equipment and software solutions in this urgent public health crisis’ by making their patents and copyright freely available to combat the pandemic.^37^ The rationale for the Open COVID Pledge can be seen from Bentham's utilitarian prism.^38^ Those who have taken the pledge have taken up the responsibility to seek the greatest good for the greatest number of people.^39^ They have chosen to prioritise the public interest above individual profit.^40^ Of course, we have the economic and incentive theories which would liken anyone who finds the cure to COVID‐19, to one who has sown seeds and deems it just to reap the pecuniary benefits of their ingenuity, labour and investment.^41^ This perspective places the individual interest above the public interest but in the face of this pandemic, the public interest must be prioritised above individual interests. Fortunately, the global community appears to be inclined to collaborations and are poised to work in the public interest.^42^

This article addresses the role of traditional medicine in the COVID‐19 battle. It situates traditional medicine within the traditional knowledge (TK) and IP discussion. It discusses the increased gravitation towards using traditional medicine but also identifies the existence of scepticism towards its use. Indigenous communities make up a large portion of the traditional medicine community and therefore called upon to play their role in the fight against COVID‐19. The battle is real, the coronavirus ground is an intersecting one requiring the exploration of all possible solutions. This article advocates for collaboration. It is a clarion call to all stakeholders to join hands to fight the virus. It advocates inclusion rather than exclusion.

### 1.3 COVID‐19 and the gravitation towards traditional Medicine

As noted earlier, science has made great strides in developing COVID‐19 vaccines. However ‘access to vaccine is an issue. The vaccine nationalist approach adopted by some nations in the global north coupled with their refusal to waive IP rights (IPRs) for COVID‐19 vaccines and personal protection equipment (PPE) is tantamount to acquiescing to the death of millions of people in the global south’.^43^ The IP related vaccine waiver contentions at the WTO is evidence of the complexities inherent in the global IP system.^44^ When members of the global south could not access the vaccine, they explored other solutions. In indigenous communities in China, India, Nigeria and Thailand, there has been widespread use of local therapies consisting of a combination of herbs and plants used both as preventive and curative methods for coronavirus.^45^ This has once again brought to the fore the debate about Traditional Medicine and the contest between traditional and western medical practitioners.^46^ Reports in both mainstream and social media were awash with accounts and recommendations for a warm mixture of garlic, ginger, lemon, black pepper and turmeric as a magic anticoronavirus cure. In Nigeria for example, the Ooni of Ife, one of the prominent traditional rulers in Nigeria, released a video promoting the use of herbal remedies to treat and prevent corona virus.^47^ In China rumours that herbal remedies could combat the virus led to panic buying of traditional medicines.^48^ In Cameroun, the traditional healers have been overwhelmed by the rush for herbal remedies.^49^ In Thailand, people rushed to buy herbal remedies in the hope that it would cure and prevent the coronavirus. CTN news reported that a traditional medicinal centre put out a notice outside their office, that the traditional herbal medicines have four antiviral properties including preventing the virus from entering the cells; reduces virus cell division; boosts immunity, and ameliorates lung inflammation from viral infection.^50^

## 2 TRADITIONAL MEDICINE AND TK

The role of traditional medicine in providing healthcare is well documented and established. Ancient civilisations such as Mesopotamia (2900 B.C.), Egypt (1500 B.C.), China (1100 B.C.), India (1000 B.C.), Greece (300 B.C.), and Rome (100 A.D.) have documented knowledge of the use of traditional medicine.^51^ ‘It is common knowledge that animals (including carnivores) often feed on certain plants, grasses or berries when they are sick. Hence, it is not surprising that since the dawn of civilisation humans have learned to use plants and plant‐derived products as remedies for various ailments… Such practices are seen in traditional cultures, often followed by village shamans or tribal medicine men’.^52^

Traditional medicines are millennia old and are an integral component of TK.^53^ They can be contrasted with western medicine which are based on scientifically proven evidence which emerged gradually from the empirical knowledge of traditional medicines in ancient civilisations (Egypt, Mesopotamia, Greece, India, and China).^54^ Western and traditional medicine converge in their objective of providing healthcare but their approach, methods and philosophies differ.^55^ Whereas traditional medicine stems from TK and focuses on both the body and mind for diagnosis, prevention and treatment, western medicine is hinged on science and ‘focuses on the suppression of symptoms on target parts of the body’.^56^ A marked difference between them is the low acceptance level of traditional medicine by the scientific community.^57^

TK also referred to as indigenous or aboriginal knowledge is described by the World Intellectual Property Organisation (WIPO), as ‘knowledge, know‐how, skills and practices that are developed, sustained and passed on from generation to generation within a community, often forming part of its cultural or spiritual identity’.^58^ TK has been recognised as a tool for sustainable development, especially among the indigenous and local communities.^59^ TK has an intrinsic communal characteristic and therefore differs from conventional IPR which is built on an individualistic authorship/ownership structure.^60^ The implication is that TK within most indigenous and local communities are not protectable under the current IP regime as they are unable to satisfy the authorship/ownership requirements.^61^ TK products are however often exploited by western countries who obtain economic interests and a recognition of IP protection for the work done exploiting the TK.^62^ National and international pressure for the protection of TK brought about an International legislative framework (Convention for Biological Diversity [CBD] and the 2010 Nagoya Protocol), that supports the recognition and protection of TK.^63^ Article 8 of the CBD requires that parties respect and maintain the TK of indigenous and local communities. It encourages the promotion of a wider application of TK and the fair and equitable sharing of benefits from the utilisation of this knowledge.^64^ Article 10 provides for the protection and encouragement of customary use of TK in accordance with traditional cultural practices, thereby encouraging the use of TK within the local communities.^65^ Article 16 recognises TK as a vital technology for effective practices of conservation.^66^ In the preamble to the Nagoya protocols it notes ‘the interrelationship between genetic resources and traditional knowledge, their inseparable nature for indigenous and local communities, the importance of the traditional knowledge for the conservation of biological diversity and the sustainable use of its components, and for the sustainable livelihoods of these communities’.^67^ The protocol provides for access to TK associated with genetic resources based on prior informed consent and the involvement of the indigenous and local communities.^68^ It is therefore not surprising that the use of traditional medicine is prevalent globally.^69^ According to the World Health Organization, 75% of the world's populations are using herbs for basic healthcare needs.^70^ The preference for natural products above western pharmaceutical products is not unconnected to data which shows that using natural products boosts the immunity of the body, cures and prevents sicknesses.^71^

The term ‘traditional medicine’ encompasses practices that use natural resources from plants, animals, nature and spiritual therapies to provide health care.^72^ Fokunang et al. describes it as ‘health practices, approaches, knowledge and beliefs incorporating plant, animal and mineral based medicines, spiritual therapies, manual techniques and exercises, applied singularly or in combination to treat, diagnose and prevent illnesses or maintain well‐being.’^73^ The above practices align with WIPO's definition of TK. These practices are based on different factors including cultural traditions that have been passed down from one generation to another. It also stems from an understanding of natural resources such as plants, animal skins, ointments and other such resources. In indigenous cultures, elders often have a special understanding of nature, herbs, plants and how this TK can help heal their people.^74^ Certain tribes and clans are bestowed with the responsibility of guarding this cultural heritage and providing traditional medical care to the vulnerable when it is required.^75^ These cultural practices and the use of native plants intersect with TK and the use of genetic resources. The use of plants for healing is not new and it is common knowledge that most drugs come from plants.^76^ The active ingredients in drugs are mostly taken from plants hence it is no surprise that herbs and traditional use of plants have been identified as products that are efficient for boosting the immune system, attacking different types of sicknesses and for preventing different types of ailments.^77^ Pharmaceutical companies leverage on the patent system, particularly the trade‐related aspects of IP to commercialise the active ingredients in plants used for traditional medicine. These commercialisations are commendable when seen from the prism of healthcare for all humanity. However, when these drugs have been manufactured, access to medicine is often restricted like was the case during the AIDS pandemic (antiretroviral drugs) and now with the Coronavirus (COVID vaccines). This has raised debates in many quarters about the imperative for access to the knowledge embedded in traditional medicine and at the same time the misappropriation of indigenous knowledge by patent holders.

In a trilateral study by WHO, WIPO, and WTO it was acknowledged that ‘The growth in the trade of health products based on traditional knowledge (TK), coupled with growth in the use of TK as a lead for biomedical research and product development, have provoked a policy debate about the misappropriation of TK and the development of, and compliance with, appropriate protocols for access to, and use of TK, especially traditional medical knowledge. The related issues of prior informed consent (PIC) and equitable benefit‐sharing (EBS), while ensuring continued R&D, have also formed part of this debate’.^78^ As noted above, the Nagoya protocol (CBD) provides a regulatory platform for addressing the trade‐related concerns sparked by the exploitation of traditional medical knowledge under the auspices of the current global patent system.

### 2.1 Scepticism about traditional medicine

Traditional medicine is practiced by over 6000 unique indigenous peoples around the world and they each have their peculiar belief systems.^79^ Available writings on the traditional medicine practices of these communities are mostly inaccurate.^80^ Hardison rightly noted that ‘This is partly because so much of this literature is written by non‐indigenous academics, who carry a number of cultural and professional assumptions into the debates. They assume the primacy of the western IP norms, and then describe indigenous knowledge in terms of that framework. This leaves some fundamental concepts of indigenous culture unexamined’.^81^

Traditional medicine was once termed primitive by western medicine. The practice of traditional medicine was often shrouded in secrecy and there was hardly any records for tracking or investigation purposes.^82^ As a result, the use of traditional medicine is often viewed with scepticism.^83^ The scientific community often frown at the idea of traditional medicine and argue that ‘Thousands of years of usage and faith cannot be taken as evidence for efficacy of traditional herbs’.^84^ The practice of traditional medicine can no more be described as primitive. It has become a structured practice with ethics and good practice.^85^ According to Ezekwesili and Okaka (2019), ‘the future of traditional medicine is bright if viewed in the context of service provision, increase of health care coverage, economic potential, and poverty reduction. Formal recognition and integration of traditional medicine into conventional medicine will hold much promise for the future’.^86^

Scepticism about traditional medicine is often scepticism about the global south. It is the perception that nothing of value can come out of the global south; that the global south is made up of the user communities while the global north is made up of innovators, manufacturers and global problem solvers.^87^ The politics that fuels these perceptions can be seen through the lens of an international treaty (Intergovernmental committee for TK, traditional cultural expression [TCE] and genetic resources [GR]) which ought to serve the interest of the global south, but which has been caught in a ubiquitous circle for about 20 years. The committee was mandated to negotiate text‐based instruments for the effective protection of GRs, TK and TCEs within the IP system.^88^ The complexities of international IP negotiations is not in question.^89^ However, 2020 makes it 20 years since the negotiations commenced and the failure to reach a unanimous decision is not only disappointing but frustrating. The frustration is unfortunately that of the global south and the status quo is apparently favourable to the global north.^90^ Oguamanam succinctly captured the essence of this politics when he noted that

For non‐demandeur country experts and member states of WIPO, the protracted delays of the WIPO IGC to agree on the text(s) of instruments arising from its mandate is perhaps less disconcerting. This is so because as non‐demandeurs (mainly countries of the Global North), they came into the negotiation with little or no vested interest in a stronger TK/TCEs regime and its interface with IP and GRs… Save for a few countries in the Global North, many others are equally reluctant and unconvinced participants in the IGC process. For these categories, the status quo is desirable, as no outcome is perhaps a better outcome.^91^

### 2.2 The Abalaka story

The suppression of any plausible solution from the global south was demonstrated in Nigeria when the HIV/AIDS pandemic was ravaging the world and about 5% of Nigerians were said to be infected with the virus.^92^ At the time, a Medical doctor, Jeremiah Abalaka claimed he had found the cure for HIV/AIDS.^93^ One would have expected that such innovation would have been supported and celebrated by the government, but instead Abalaka's treatment was banned. The Nigerian Vice President alleged that the vaccine had killed more than it had cured and therefore suspended the treatment and the use of all similar HIV/AIDS therapies.^94^ This action was met with public protests. The protester alleged that the government was colluding with international pharmaceutical giants to frustrate the efforts of Abalaka.^95^ They staged peaceful demonstrations, calling on the President to dismiss the Minister of Health for colluding with the international community to sabotage Abalaka's vaccine program.^96^ In a 2014 lawsuit filed by Dr Abalaka against the President of the Federal Republic of Nigeria, the Attorney General and the National Agency for Food and Drug Administration (NAFDAC),^97^ the plaintiff challenged the justification of the ban on his vaccine. The Federal High Court held in the plaintiff's favour declaring the ban on Abalaka's vaccine arbitrary, illegal, null, and void. It noted that since the plaintiffs' vaccine is a drug, patients' consent must be obtained before the administration of the vaccine.^98^ The influence of the global north on the government of the global south to frustrate Abalaka's innovation ties into the perception that nothing of value can come out of the global south and that the global south is merely a user community, while the global north are the innovators.^99^

### 2.3 Global south and global north

It is one thing for the global north to frustrate the global south due to the developmental divide, but when the global north succeeds in using the leadership of the global south to frustrate innovations coming from their citizens, it becomes unfortunate, and in Chinua Achebe's words, things fall apart and the centre cannot hold.^100^ An example of the global north's negative influence on the global south is the intimidation of the United States on South Africa regarding the Draft Copyright Amendment Bill.^101^ Unfortunately, it is the commoner (the members of the global south) that suffer the tragedy. The expectation from the leadership of the global south is for them to encourage and provide enabling opportunities for development rather than banning and discouraging their citizens. Most developing countries are not known for discovering ground‐breaking treatments. One will therefore think that a story like Abalaka's, would see the government providing support and encouragement. This is what is required, and it is how development has thrived in other parts of the world. Without an enabling environment, development becomes stifled.^102^ Michael J. Finger rightly noted that the question we need to ask is ‘How can we help poor people to earn more from their knowledge–it is by promoting the innovation, knowledge, and creative skills of poor people in poor countries’^103^ He also noted that ‘the normal commercial and legal instruments that work well in richer societies can work for poorer people’.^104^

## 3 THE WAY FORWARD

The CBD and Nagoya Protocol encourages the use of traditional medicine.^105^ Many indigenous and local communities have awoken to the effectiveness of their indigenous practices for purposes of healthcare and it is working for them.^106^ In 1996, Clement, a western‐styled accountant but also a member of an indigenous local community was involved in a serious motor accident and was rushed to the hospital. He fractured the bones in his left hand and was told by the doctors that he would have to amputate the hand because it was hopeless. Someone directed him to a traditional/herbal practitioner who was skilled in rearranging bones. The traditional herbal healer assured him that he had seen and treated worse cases. He was treated with herbs, leaves and ointments and after about 4 months, the use of his hand was restored. It's been over 24 years now and Clement's hand is still in excellent shape.

Many members of western communities have embraced the use of traditional medicine by using herbal and natural products.^107^ The coronavirus pandemic is reminding the world that traditional medicine preceded western medicine and that the TK passed down from generation to generation on how to diagnose, prevent and treat sick people is an effective mean for our healthcare problems.^108^ The indigenous and local communities therefore have a responsibility to draw on this knowledge and help the world at this precarious time. It could be that the solution to addressing the problems associated with the coronavirus has been passed down from our ancestors and that the answers to the coronavirus is right with us. There is therefore a duty on the indigenous and local communities to look inward and see how to join hands in combatting this pandemic. It is also imperative that the scientific community recognise the role of traditional medicine in the battle against COVID‐19, and that they ensure that they do not ignore the cure because of bias and scepticism.

It is not correct to think or say that the global south is merely a ‘user community’ and do not have a productive role to play in the global search for a cure.^109^ Governments of the global south should launch ambitious private and public sector collaborative research initiative with the goal of finding a cure for coronavirus using local resources just like the developed countries are looking for vaccines. Why should we think that the global south does not have a productive role to play in the global search for a cure? Success lies in the hands of those who dare to try. Once, a prominent IP scholar asked whether ‘Creativity has died in the third world?’.^110^ There is no doubt that it has not died, only that it has flourished and COVID provides a great opportunity for Traditional Medicinal Knowledge.

## 4 CONCLUSION

The battle against this virus should not be seen as a competition (who will be the first to find the cure) but rather as a rescue mission (let us save the vulnerable). This mindset is imperative because the goal is to work in the public interest, that is, the greatest good for the greatest number of people. The coronavirus is a global pandemic that requires collaborative efforts. The global north is working hard, and their governments and funding organisations are providing the needed support. The global south also has a role to play and should be active in this search either via western or traditional medicine. The traditional medicine communities (made up of indigenous local communities) must also join hands to find a cure. This article is a clarion call to all stakeholders, governments and funding organisations to support the search for a cure, be it through western or traditional medicine. No one should throw spanners in the wheel of progress. What is required is support and encouragement. It is important for guidelines to be adhered to, but scepticism must not be allowed to debar the possible contributions from traditional medicine and from indigenous and local communities. The global south should rally around themselves and not allow external influences frustrate internal efforts. The call is for all interest groups to collaborate towards the same goal because we achieve more when we are together than when we are divided.

## CONFLICT OF INTEREST

The author declares no conflict of interest.

## Data Availability

The data that support the findings of this study are available upon reasonable request from the corresponding author

## ENDNOTES

1 Virander Chauhan, ‘The Race to Find a Cure for COVID‐19’ (*The Hindu* 2020) <https://www.thehindu.com/opinion/op-ed/the-race-to-find-a-cure-for-COVID-19/article31156509.ece> accessed 25 March 2020.

2 John Modell Timothy Haggerty, ‘The Social Impact of War’ (1991) 17(1) ARS 205.

3 Mridula Amin, ‘NSW Healthcare Workers Struggling To Keep Families Safe From Coronavirus’ (*ABC News*, 2020) <https://www.abc.net.au/news/2020-04-02/nsw-healthcare-workers-leaving-families-in-coronavirus-crisis/12111218> accessed 2 April 2020.

4 Isabella Kwai, Australia's First Recession in Decades Signal Tough Times to Come (*The New York Times* 2020) <https://www.nytimes.com/2020/09/02/business/australia-recession.html> accessed 4 September 2020.

5 Ibid.

6 Szu P. Chan, ‘Coronavirus: World Faces Worst Recession Since Great Depression’ (Business Reporter, *BBC News* 2020) <https://www.bbc.com/news/business-52273988> accessed 4 September 2020.

7 Ibid.

8 World Health Organisation ‘Rolling Updates on Coronavirus Disease (COVID‐19)’ (Web Page) <https://www.who.int/emergencies/diseases/novel-coronavirus-2019/events-as-they-happen#:%7E:text=The%20outbreak%20was%20declared%20a,Concern%20on%2030%20January%202020.%26text=On%2011%20February%202020%2C%20WHO,coronavirus%20disease%3A%20COVID%2D19> accessed 17 June 2020.

9 World Health Organisation, ‘WHO Timeline COVID‐19’ (Web Page) <https://www.who.int/news-room/detail/27-04-2020-who-timeline---COVID-19> accessed 27 April 2020.

10 Worldometer, *‘Coronavirus cases*’ (Web Page) <https://www.worldometers.info/coronavirus/?utm_campaign=homeAdvegas1?> accessed 25 June 2020.

11 Paral Goyal and Others, ‘Clinical Characteristics of COVID‐19 in New York City’ (2020) 382 NEJM 2372. <https://doi.org/10.1056/NEJMc2010419>

12 Reed Albergotti and Faiz Siddiqui, ‘Ford and GM are Undertaking a Warlike Effort to Produce Ventilators. It May Fall Short and Come Too Late: Inside The Auto Giants’ Efforts to Build Prototypes and Revamp Factories to Produce Tens of Thousands of Ventilators In Time for COVID‐19 Patients' *The Washington Post*
<https://www.washingtonpost.com/business/2020/04/04/ventilators-coronavirus-ford-gm/> accessed 5 April 2020.

13 Letter from Chancellor of the Duchy of Lancaster to Chair of Public Accounts Committee (PAC), Reference MC2020/03231. <https://www.gov.uk/government/publications/COVID-19-contingent-liability-for-ventilators--2>

14 Joshua Geleris, ‘Observational Study of Hydroxychloroquine in Hospitalized Patients with COVID‐19’ (2020) 382 NEJM 2411. <https://doi.org/10.1056/NEJMoa2012410>

15 Brad Ryan, ‘Hydroxychloroquine, used by Donald Trump, to be Trialled by Australian Health Workers against Coronavirus’ *Australian Broadcasting Corporation* <https://www.abc.net.au/news/2020-05-20/coronavirus-hydroxychloroquine-trial-donald-trump-game-changer/12266398> accessed 21 May 2020.

16 Soutik Biswas, ‘Hydroxychloroquine: The Unproven “Corona Drug” Trump is Threatening India for’ *BBC News* <https://www.bbc.com/news/world-asia-india-52180660> accessed 7 April 2020.

17 Ibid.

18 Joe Pierre, ‘Why Aren't Some People Taking COVID‐19 More Seriously? Fear‐Based Risk Assessments, Misinformation and Conspiracy Theories’ *Psychology Today* <https://www.psychologytoday.com/au/blog/psych-unseen/202003/why-arent-some-people-taking-COVID-19-more-seriously> accessed 26 March 26 2020.

19 International labour Organisation, ‘COVID‐19 Causes Unprecedented Jobs Crisis, Almost All Workers And Businesses Affected By Lockdown Measures’ Web page <https://www.ilo.org/global/about-the-ilo/newsroom/news/WCMS_748441/lang--en/index.htm> accessed 19 June 2020.

20 ‘COVID‐19 fear fuels rush for traditional herbal medication: ‘No scientific evidence’ that herb is effective, cautions doctor at Chaophraya Abhaibhubejhr Hospital’ *Bangkok Post* <https://www.bangkokpost.com/thailand/general/1880490/COVID-19-fear-fuels-rush-for-traditional-herbal-medication> accessed 17 March 2020.

21 Godwin Anywar and Others, ‘Medicinal Plants Used by Traditional Medicine Practitioners to Boost The Immune System in People Living with HIV/AIDS in Uganda’ (2020) 3(35) EJIM 35, 101011 <https://doi.org/10.1016/j.eujim.2019.101011>

22 Timothy Dyke and Warwick P. Anderson, ‘A History of Health and Medical Research in Australia’ (2014) 201 (1) MJA 33. <https://doi.org/10.5694/mja14.00347>

23 Orenstein Walter and Rafi Ahmed, ‘Simply Put: Vaccination Saves Lives’ (2017) 114(16) PNAS USA 4031. <https://doi.org/10.1073/pnas.1704507114>

24 Weatherall D and Others, ‘Science and Technology for Disease Control: Past, Present, and Future’ in Jamison DT et al. (eds), *Disease Control Priorities in Developing Countries* (Oxford University Press and World Bank, 2006).

25 Gupta Ranjan and Others ‘Nature's Medicines: Traditional Knowledge and Intellectual Property Management. Case studies from the National Institutes of Health (NIH), USA’ (2005) 2(4) CDDT 203. <https://doi.org/10.2174/157016305775202937>

26 Emilie Cloatre, ‘Law and Biomedicine and the Making of “Genuine” Traditional Medicines in Global Health’ (2019) 29(4) CPH 424. <https://doi.org/10.1080/09581596.2019.1594696>

27 Kamboj VP, ‘Herbal Medicine’ (2000) 78(1) CS 35.

28 Junhua Zhang and Others, ‘The Safety of Herbal Medicine: From Prejudice to Evidence’ (*Hindawi* 2015) <https://doi.org/10.1155/2015/316706>

29 John Elflein, ‘New Cases of COVID‐19 Worldwide from January 23 to June 22, 2020’ *Statista* <https://www.statista.com/statistics/1103046/new-coronavirus-COVID19-cases-number-worldwide-by-day/> accessed 24 June 2020.

30 Scott Morrison, ‘Press Conference: Australian Parliament House, ACT’ *Press Release* <https://www.pm.gov.au/media/press-conference-australian-parliament-house-act-250320> accessed 25 March 2020,

31 The Holy Bible, Genesis 1:28.

32 Jonathan Gardner, Ned Pagliarulo, and Ben Fidler, ‘Coronavirus Vaccines are Rolling Out Quickly. Here's Where The Pipeline Stands <https://www.biopharmadive.com/news/coronavirus-vaccine-pipeline-types/579122/> accessed 27 May 2021)

33 Antoinette Baujard, ‘Collective Interest versus Individual Interest in Bentham's Felicific Calculus. Questioning welfarism and fairness’ (2010) 17(4) EJHET 607.

34 Sun Tzu, *The Art of War*, (Canterbury, 2014) 30–31.

35 Ibid.

36 National Institute of Health, COVID‐19 is an Emerging Rapidly Evolving Situation <https://ncats.nih.gov/COVID19-translational-approach/collaboration> accessed 4 September 2020.

37 Creative Commons, Open COVID Pledge: About Us <https://openCOVIDpledge.org/about/> accessed 4 September 2020.

38 See generally, Rosenblum Nancy, *Bentham's Theory of the Modern State* (Cambridge University Press, 1978)

39 Julia Janewa Osei Tutu, ‘Humanizing Intellectual Property: Moving Beyond the Natural Rights Property Focus’ (2017) 20 VJETL 244.

40 William Fisher, ‘Theories of Intellectual Property’ in Stephen R. Munzer (ed), *New Essays in the Legal and Political Theory of Property* (Cambridge University 2001) 168, 169–170.

41 Peter Drahos, *A Philosophy of Intellectual Property* (ANU Press 2016) 33.

42 United Nations, ‘Landmark collaboration’ to make COVID‐19 testing and treatment available to all, UN News <https://news.un.org/en/story/2020/04/1062512> accessed 24 April 2020. World leaders, scientists, humanitarians and private sector partners united on Friday in a “landmark collaboration” that will make new tools and medicines to diagnose and treat COVID‐19, accessible to people everywhere. See also, World Health Organisation, Public statement for collaboration on COVID‐19 vaccine development <https://www.who.int/news-room/detail/13-04-2020-public-statement-for-collaboration-on-COVID-19-vaccine-development> accessed 13 April 2020. We are scientists, physicians, funders and manufacturers who have come together as part of an international collaboration, coordinated by the World Health Organization (WHO), to help speed the availability of a vaccine against COVID‐19. While a vaccine for general use takes time to develop, a vaccine may ultimately be instrumental in controlling this worldwide pandemic. In the interim, we applaud the implementation of community intervention measures that reduce spread of the virus and protect people, including vulnerable populations, and pledge to use the time gained by the widespread adoption of such measures to develop a vaccine as rapidly as possible. We will continue efforts to strengthen the unprecedented worldwide collaboration, cooperation and sharing of data already underway. We believe these efforts will help reduce inefficiencies and duplication of effort, and we will work tenaciously to increase the likelihood that one or more safe and effective vaccines will soon be made available to all.

43 Kunle Ola, ‘Covid‐19 Strategic Global North‐South Divide: Access to Knowledge Quadrants’ (2021) 43(12) EIPR 776.

44 Ruth L Okediji, ‘With a Covid‐19 Vaccine Patent Waiver Likely, Time To Rethink Global Intellectual Property Rules’ <https://edition.cnn.com/2021/05/07/opinions/covid-vaccine-patent-waiver-as-equals-intl-cmd/index.html> accessed 11 January 2022

45 Moki Edwin Kindzeka, ‘Cameroon's Traditional Healers See Rush for Herbal Medicines to Treat COVID‐19’ <https://www.voanews.com/africa/cameroons-traditional-healers-see-rush-herbal-medicines-treat-COVID-19> accessed 13 May 2020.

46 Kamboj (n 27).

47 Adesina Omoyooba, ‘A King's Belief in Herbal Remedies to cure COVID‐19 Butts Up against Science in Nigeria’ *Global Voices* <https://globalvoices.org/2020/04/01/a-kings-belief-in-herbal-remedies-to-cure-COVID-19-butts-up-against-science-in-nigeria/> accessed 1 April 2020.

48 AFP‐JIJI, ‘Herbal Remedies for the Coronavirus Spark Debate over Traditional Chinese Medicine’ *The Japanese Times* <https://www.japantimes.co.jp/news/2020/02/03/asia-pacific/science-health-asia-pacific/herbal-remedies-coronavirus-traditional-chinese-medicine/#.XvHfnmgzaK0> accessed 3 April 2020.

49 Moki E. Kindzeka, ‘Cameroon's Traditional Healers See Rush for Herbal Medicines to Treat COVID‐19’ <https://www.voanews.com/africa/cameroons-traditional-healers-see-rush-herbal-medicines-treat-COVID-19> accessed 13 May 2020.

50 CTN News, ‘Thai Rush to Buy Traditional Medicinals and Herbs to Fight Virus’ <https://www.chiangraitimes.com/health/thai-rush-to-buy-traditional-medicinals-and-herbs-to-fight-virus/> accessed 18 March 2020).

51 Lesley Smith, ‘The Kahun Gynaecological Papyrus: ancient Egyptian medicine’ (2011) 37 JFPRHC 5455.

52 Gupta (n 25) 203–219.

53 Margaret Bruchac, ‘Indigenous Knowledge and Traditional Knowledge’ in Smith C. (Ed) *Encyclopedia of Global Archaeology*, (Springer, 2014) 3814–3824.

54 Lemonnier and Others, ‘Traditional Knowledge‐based Medicine: A Review of History, Principles, and Relevance in the Present Context of P4 Systems Medicine’ (2017) 2(7) PPM. <https://doi.org/10.1097/pp9.0000000000000011>

55 Ibid.

56 Ibid.

57 Ibid.

58 World Intellectual Property Organisation, *Traditional Knowledge* <https://www.wipo.int/tk/en/tk/> accessed 23 June 2020.

59 Freedom‐KaiPhillips, ‘Intellectual Property Rights in Traditional Knowledge: Enabler of Sustainable Development’ (2016) 32(83) UJIEL 1. <https://doi.org/10.5334/ujiel.283>

60 Candra Irawan, ‘Protection of Traditional Knowledge: A Perspective on Intellectual Property Law in Indonesia’ (2017) 1–2 JWIP 57 <https://doi.org/10.1111/jwip.12073>

61 TRIPS: Agreement on Trade‐Related Aspects of Intellectual Property Rights, Apr. 15, 1994, Marrakesh Agreement Establishing the World Trade Organization, Annex 1C. Part II. (hereinafter TRIPS Agreement).

62 Phillips (n 59).

63
*Convention on Biological Diversity*, United Nations 1992 (‘Convention on Biological Diversity’). See also The *Nagoya Protocol on Access to Genetic Resources and the Fair and Equitable Sharing of Benefits arising from their Utilization to the Convention on Biological Diversity*, 29 October 2010 (‘Nagoya Protocol’).

64 Convention on Biological Diversity, Art 8J.

65 Convention on Biological Diversity, Art 10C.

66 Convention on Biological Diversity.

67 Nagoya Protocol.

68 Nagoya Protocol Art 7.

69 Pan Si‐Yuan and Others, ‘Historical Perspective of Traditional Indigenous Medical Practices: The Current Renaissance and Conservation of Herbal Resources’ (2014) ECcAlM. <https://doi.org/10.1155/2014/525340>

70 World Health Organisation, ‘WHO Launches the First Global Strategy on Traditional and Alternative Medicine’ *Media Centre* <https://www.who.int/mediacentre/news/releases/release38/en/> accessed 16 May 2002.

71 MS Donaldson, ‘Nutrition and Cancer: A Review of the Evidence for an Anti‐cancer Diet’ (2004) 3 NJ 19. <https://doi.org/10.1186/1475-2891-3-19>

72 Gupta (n 25) 203–219.

73 CN Fokunang and Others, ‘Traditional Medicine: Past, Present and Future Research and Development Prospects and Integration in the National Health System of Cameroon’ (2011) (8)3 AJTCAM 284–295. <https://doi.org/10.4314/ajtcam.v8i3.65276>

74 Tabona Shoko, *Karanga Indigenous Religion in Zimbabwe: Health and Well‐Being* (Routledge, 2016) 48.

75 Molly Torsen and Jane Anderson, ‘Intellectual Property and The Safeguarding of Traditional Cultures: Legal Issues and Practical Options for Museums, Libraries and Archives’ (World Intellectual Property Organization (WIPO), 2010). <https://www.wipo.int/edocs/pubdocs/en/tk/1023/wipo_pub_1023.pdf>

76 C Katiyar, A Gupta, S Kanjilal, and S Katiyar, ‘Drug Discovery From Plant Sources: An Integrated Approach’ (2012) 33(1) Ayu 10 <https://doi.org/10.4103/0974-8520.100295>

77 Donaldson (n 71).

78 WTO, Promoting Access to Medical Technologies and Innovation Intersections between public health, intellectual property and trade in Intellectual Property: WHO‐WIPO‐WTO Book <https://www.wto.org/english/tratop_e/trips_e/trilatweb_e/trilat_web_13_e.htm>

79 Preston Hardison, ‘Indigenous People and the commons’ *On the Commons* <http://www.onthecommons.org/indigenous-peoples-and-commons#sthash.owu2EsG2.dpbs> accessed 20 November 2006.

80 Ibid.

81 Ibid.

82 Ezekwesili‐Ofili Josephine Ozioma and Okaka Antoinette Nwamaka Chinwe, ‘Herbal Medicines in African Traditional Medicine’ (2019) *IntechOpen*. <https://doi.org/10.5772/intechopen.80348>. <https://www.intechopen.com/books/herbal-medicine/herbal-medicines-in-african-traditional-medicine>

83 Pan Si‐Yuan (n 69)

84 Yichang Yang, ‘Use of Herbal Drugs to Treat COVID‐19 Should be With Caution’ (2020) 395(10238) Lancet 1689.

85 Chatfield Kate et al. ‘Applying an Ethical Framework to Herbal Medicine’ (2018) EBCAM. <https://doi.org/10.1155/2018/1903629>

86 Ezekwesili‐Ofili Josephine Ozioma and Okaka Antoinette Nwamaka Chinwe (n 82).

87 Lemuel Ekedegwa Odeh, ‘A Comparative Analysis of Global North and Global South Economies’ (2010) 12(3) JSDA 341.

88 Chidi Oguamanam, ‘Tiered or Differentiated Approach to Traditional Knowledge and Traditional Cultural Expressions: The Evolution of a Concept’ (2018) CIGI, 1‐30, 5.

89 Peter K Yu, ‘International Enclosure, the Regime Complex, and Intellectual Property Schizophrenia’ (2007) *Mich St L Rev* 1–33.

90 See generally, Daniel F Robinson, Ahmed Abdel‐Latif & Pedro Roffe, eds, *Protecting Traditional Knowledge: The WIPO Intergovernmental Committee on Intellectual Property and Genetic Resources, Traditional Knowledge, and Folklore* (Routledge 2017).

91 Chidi Oguamanam (n 88).

92 Barnaby Phillips, ‘Nigerian Doctor finds HIV Cure’ *BBC News* <http://news.bbc.co.uk/2/hi/africa/740523.stm> accessed 8 May 2020.

93 Obadare, Ebenezer, and Iruka N. Okeke. ‘Biomedical Loopholes, Distrusted State, and the Politics of HIV/AIDS ‘Cure’ in Nigeria’ (2011) 110(439) AA 191–211. JSTOR <https://www.jstor.org/stable/41240189>.

94 Ahmad K, ‘Public protests as Nigeria Bans Use of Untested HIV Vaccine’ (2000) 356(9228) *Lancet* 493. <https://doi.org/10.1016/S0140-6736(05)74166-0>

95 Ibid.

96 Ibid.

97
*Dr. Jeremiah Ojonemi Alabi Abalaka and Medicrest Specialist Hospital Ltd*. v. *President of the Federal Republic of Nigeria, Attorney General of the Federation, and National Agency for Food and Drug Administration and Control (NAFDAC)* [2014] Suit No. FHC/MKD/CS/75/2012 Nigeria, Federal High Court, Makurdi.

98 Ibid.

99 Lemuel Ekedegwa Odeh, ‘A Comparative Analysis of Global North and Global South Economies’ (2010) 12(3) JSDAf, 341.

100 Chinua Achebe, *Things Fall Apart* (Heinemann, 1996) iix.

101 Sean Flynn, ‘Making sense of South Africa's new copyright bill and US trade threats’ *The Conversation* <https://theconversation.com/making-sense-of-south-africas-new-copyright-bill-and-us-trade-threats-128418> accessed 15 December 2019.

102 Ola Kunle, *Open Access to Knowledge in Nigeria: A Framework for Developing Countries* (Routledge, 2018) 216.

103 Michael J Finger and Philip Schuler (eds), *Poor People's Knowledge, Promoting Intellectual Property in Developing Countries* (Oxford University Press, 2004) 1.

104 Ibid.

105 See *Convention on Biological Diversity* and *The Nagoya Protocol*

106 Tabona (n 74).

107 Kamboj (n 27).

108 Lemonnier et al. (n 54).

109 Rory Horner, Seth Schindler, Daniel Haberly, and Yuko Aoyama, ‘Globalisation, uneven development and the North–South “Big Switch”’ (2018) 11(1) CJRES 17. <https://doi.org/10.1093/cjres/rsx026>

110 Ruth L. Gana, ‘Has Creativity Died in the Third World—Some Implications of the Internationalization of Intellectual Property’ (1995) 24 DJILP 109.

